# Treatment With Lisinopril Prevents the Early Progression of Glomerular Injury in Obese Dahl Salt-Sensitive Rats Independent of Lowering Arterial Pressure

**DOI:** 10.3389/fphys.2021.765305

**Published:** 2021-12-17

**Authors:** Andrea K. Brown, Alyssa Nichols, Chantell A. Coley, Ubong S. Ekperikpe, Kasi C. McPherson, Corbin A. Shields, Bibek Poudel, Denise C. Cornelius, Jan M. Williams

**Affiliations:** ^1^Department of Pharmacology and Toxicology, University of Mississippi Medical Center, Jackson, MS, United States; ^2^Department of Emergency Medicine, University of Mississippi Medical Center, Jackson, MS, United States

**Keywords:** ACE inhibitor, obesity, proteinuria, GFR, cytokines, SS rat, SSLepRmutant rat

## Abstract

Recently, we reported that obese Dahl salt-sensitive leptin receptor mutant (SS^LepR^mutant) rats develop glomerular injury and progressive proteinuria prior to puberty. Moreover, this early progression of proteinuria was associated with elevations in GFR. Therefore, the current study examined whether treatment with lisinopril to reduce GFR slows the early progression of proteinuria in SS^LepR^mutant rats prior to puberty. Experiments were performed on 4-week-old SS and SS^LepR^mutant rats that were either treated with vehicle or lisinopril (20 mg/kg/day, drinking water) for 4 weeks. We did not observe any differences in MAP between SS and SS^LepR^mutant rats treated with vehicle (148 ± 5 vs. 163 ± 6 mmHg, respectively). Interestingly, chronic treatment with lisinopril markedly reduced MAP in SS rats (111 ± 3 mmHg) but had no effect on MAP in SS^LepR^mutant rats (155 ± 4 mmHg). Treatment with lisinopril significantly reduced proteinuria in SS and SS^LepR^mutant rats compared to their vehicle counterparts (19 ± 5 and 258 ± 34 vs. 71 ± 12 and 498 ± 66 mg/day, respectively). Additionally, nephrin excretion was significantly elevated in SS^LepR^mutant rats versus SS rats, and lisinopril reduced nephrin excretion in both strains. GFR was significantly elevated in SS^LepR^mutant rats compared to SS rats, and lisinopril treatment reduced GFR in SS^LepR^mutant rats by 30%. The kidneys from SS^LepR^mutant rats displayed glomerular injury with increased mesangial expansion and renal inflammation versus SS rats. Chronic treatment with lisinopril significantly decreased glomerular injury and renal inflammation in the SS^LepR^mutant rats. Overall, these data indicate that inhibiting renal hyperfiltration associated with obesity is beneficial in slowing the early development of glomerular injury and renal inflammation.

## Introduction

Obesity has emerged as an epidemic and major health problem over the last few decades and has been linked to the increasing prevalence of renal disease (Chagnac et al., 2000; Bosma et al., 2004; Ejerblad et al., 2006; Hsu et al., 2006; Jacobs et al., 2010). One of the major reasons for this association is that obesity is associated with the two most common causes of renal disease, hypertension and diabetes (Chen et al., 2004; Kurella et al., 2005). However, obesity alone is now considered an independent risk factor for renal injury and ultimately leads to chronic kidney disease (CKD) (Chagnac et al., 2000; Bosma et al., 2004; Ejerblad et al., 2006; Hsu et al., 2006; Jacobs et al., 2010). While there have been plenty of studies investigating the pathophysiology of renal disease in obese adults, studies examining the relationship between renal disease and obese children have been few and far between. Recent studies suggest that childhood obesity is associated with the increased risk of proteinuria in children independent of diabetes and hypertension (Ogden et al., 2016; Hales et al., 2017) indicating that renal dysfunction starts long before elevations in blood glucose levels and arterial pressure. Recently, we reported that the obese Dahl salt-sensitive (SS) leptin receptor mutant (SS^LepR^mutant) rat develops progressive proteinuria in the absence of hyperglycemia and elevations in arterial pressure prior to puberty (McPherson et al., 2016, 2020; Poudel et al., 2020). Therefore, the SS^LepR^mutant rat offers the ability to study the mechanisms involved in the early progression of renal injury associated with obesity.

One of the hallmark characteristics that contributes to renal injury in adult obese patients is elevations in GFR, also known as renal hyperfiltration (Chagnac et al., 2000; Henegar et al., 2001; Hall et al., 2004). Yet, studies examining the early changes in renal hemodynamics in obese children are limited. The renin-angiotensin system (RAS) plays important role in regulating GFR (Hall, 1986, 1991). Angiotensin II (AngII), one of the major metabolites of RAS, is elevated in obese subjects with renal disease and causes hypertension and stimulates renal inflammation (Suzuki et al., 2003; Schmieder et al., 2007; Yvan-Charvet and Quignard-Boulange, 2011; Kalupahana et al., 2012; Kalupahana and Moustaid-Moussa, 2012; Alique et al., 2014; Li et al., 2015). Moreover, RAS contributes to the early elevations in GFR during the initial stages of renal disease in obese patients (Ribstein et al., 1995; Zhang and Reisin, 2000; Price et al., 2002). We recently observed that the early progression of proteinuria in obese SS^LepR^mutant rats was associated with renal hyperfiltration (McPherson et al., 2020). Since angiotensin-converting enzyme inhibitors are one of the standard treatments for patients with albuminuria (Agodoa et al., 2001; Jafar et al., 2001; Progression of Chronic Kidney Disease, 2003; Chu et al., 2021), we hypothesized that ACE inhibition would reduce hyperfiltration and renal inflammation leading to a reduction in the early progression of proteinuria in SS^LepR^mutant rats prior to puberty.

## Materials And Methods

### General

Experiments were performed on a total of 101 SS and SS^LepR^mutant female and male rats between 4–8 weeks of age prior to puberty (Shields et al., 2021). SS and SS^LepR^mutant rats were obtained from our in-house colony of heterozygous SS^LepR^mutant rats, created by using zinc-finger nuclease technology as previously described (McPherson et al., 2016). We have previously observed the development of renal injury is similar in female and male SS and SS^LepR^mutant rats during this age (Poudel et al., 2018; Shields et al., 2021). Genotyping was performed by the Molecular and Genomic Facility at the University of Mississippi Medical Center. Rats were fed a 1% NaCl diet (TD58640; Harlan Laboratories, Madison, WI) and had free access to food and water except during the 2-h period of the GFR measurement. Rat housing in the Laboratory Animal Facility at University of Mississippi Medical Center was approved by the American Association for the Accreditation of Laboratory Animal Care, and all protocols were approved by the University of Mississippi Medical Center Institutional Animal Care and Use Committee.

### Effects of Lisinopril on the Early Progression of Renal Injury in SS and SS^LepR^mutant Rats

At 4 weeks of age, SS and SS^LepR^mutant rats were weighed and blood samples were collected *via* tail vein for measurement of blood glucose levels (glucometer, Bayer HealthCare; Mishawaka, IN). Then, the rats were placed in metabolic cages for an overnight urine collection to determine proteinuria using the Bradford method (Bio-Rad Laboratories; Hercules, CA). After collecting baseline data, SS and SS^LepR^mutant rats were separated into four groups: (1) SS and (2) SS^LepR^mutant rats treated with vehicle and (3) SS and (4) SS^LepR^mutant rats treated with lisinopril (20 kg/mg/day, in the drinking water; 16,833; Cayman Chemical Company, Ann Harbor, MI) for 4 weeks. We measured the water intake weekly to ensure that the rats were receiving the appropriate dose of lisinopril. Every 2 weeks rats were placed in metabolic cages until the rats reached 8 weeks of age, and proteinuria and blood glucose levels were measured at each time period. Nephrin excretion was measured on the final urine sample (NBP2-76751, Novus Biologicals, Littleton, CO). During the final week of the study, the rats were placed under anesthesia, and catheters were inserted into the carotid artery and jugular vein for the measurement of mean arterial pressure (MAP) and infusion of FITC-sinistrin (measurement of GFR), respectively. After a 24-h recovery period, catheters were connected to pressure transducers (MLT0699, ADInstruments, Colorado Springs, CO) coupled to a computerized PowerLab data-acquisition system (ADInstruments) to obtain conscious MAP from the rats. After a 30-min equilibration period, MAP was recorded continuously for 30 min. Immediately after measuring MAP, the jugular vein catheter was flushed with heparinized saline.

### Measurement of GFR *via* FITC-Sinistrin

After a 24-h recovery period from measuring MAP, rats were anesthetized briefly with isoflurane for assembly of the noninvasive clearance kidney device (MediBeacon, Mannheim, Germany) consisting of two light-emitting diodes that excite FITC-sinistrin (FTC-FS001; MediBeacon, Mannheim, Germany) at 480 nm, a photodiode that emits light at 531 nm, a microprocessor, and a battery. The device was attached to the back of the rat by a double-sided adhesive patch (MediBeacon, Mannheim, Germany) and secured with a rodent jacket to a region (~3 cm) on the back of the rat from which hair had been removed with a depilation cream. Rats were allowed to recover in separate cages, and a baseline measurement for 15 min was recorded. Next, a bolus injection of FITC-sinistrin (5 mg/100 g body wt, prepared as 15 mg/ml in sterile isotonic saline) was administered *via* the jugular vein followed by a bolus injection of sterile saline. During a 2-h period after bolus injection, excretion kinetics of FITC-sinistrin were measured transcutaneously at a sampling rate of 60 measurements/min with an excitation time of 10 ms/measurement and used to calculate the elimination half-life (t_1/2_) of FITC-sinistrin using a one-compartment model with MDPLab evaluation software (MediBeacon), as previously described (Pill et al., 2005, 2006; Schock-Kusch et al., 2009). GFR was determined from the t_1/2_ of FITC-sinistrin with a validated empirically derived conversion factor, as previously described (Yu et al., 2007; Schock-Kusch et al., 2009, 2011). Then, the rats were anesthetized and terminal blood samples were taken from the abdominal aorta for the measurement of plasma total cholesterol concentrations determined by ELISA (Cayman Chemical Company, Ann Arbor, MI). Both kidneys were weighed. The right kidney was cut in half, in which one half was fixed in a 10% buffered formalin solution for histology, and the other half was snapped frozen in liquid nitrogen and stored at −80°C. Renal cytokines were measured using a Bio-Plex Pro Rat Cytokine Plex Assay Reagent Kit on a Bio-Rad Bioplex 200 System according to the manufacture’s protocol (Bio-Rad Laboratories; Hercules, CA).

### Measurement of Glomerular Injury in SS and SS^LepR^mutant Rats

Paraffin kidney sections were prepared from the right kidneys collected from SS and SS^LepR^mutant rats treated with vehicle and lisinopril. Kidney sections were cut into 3 μm sections and stained with Periodic acid-Schiff (PAS). To determine glomerular injury, thirty glomeruli per PAS section from each rat were captured using a SeBa microscope equipped with a color camera (Laxco Inc., North Creek, Washington) and scored in a blinded fashion on a 0–4 scale with 0 representing a normal glomerulus, 1 representing a 25% of loss, 2 representing a 50% loss, 3 representing a 75% loss, and 4 representing >75% loss of capillaries in the tuft.

### Statistical Analysis

These data are presented as mean values ± SEM. Statistical analysis was performed using GraphPad Prism 8 (GraphPad Software, San Diego, CA). Two-way ANOVA followed by Tukey’s multiple comparisons test was used to determine the significant difference in mean values for a single time point. Time course changes in proteinuria were compared between and within SS and SS^LepR^mutant strains treated with either vehicle or lisinopril using a repeated measures three-way ANOVA followed by the Holm-Sidak test. A value of p of <0.05 was considered significantly different. The power of the studies was not enough to detect sex differences, so female and male rats were graphed together. Female rats in each group are represented by partially filled symbols.

## Results

### Comparisons of Metabolic Parameters

Measurement of body weight, blood glucose, plasma total cholesterol levels in SS and SS^LepR^mutant rats treated with either vehicle or lisinopril are presented in Table 1. At the end of the study, body weight was significantly higher in SS^LepR^mutant rats compared to SS rats treated with vehicle (365 ± 15 and 276 ± 16 g, respectively), and treatment with lisinopril had no effect on body weight in either strain (388 ± 13 and 245 ± 10 g, respectively). Non-fasting blood glucose levels were similar in all groups and within normal physiological range (≤120 mg/dl). Plasma total cholesterol levels were markedly elevated in vehicle-treated SS^LepR^mutant rats versus SS rats (449 ± 27 vs. 175 ± 31 mg/dl, respectively). After chronic treatment with lisinopril, plasma total cholesterol was significantly reduced in SS^LepR^mutant rats (314 ± 35 mg/dl) but not in SS rats (164 ± 15 mg/dl).

**Table 1:** Comparison of metabolic parameters in vehicle and lisinopril-treated SS and SS^LepR^mutant rats at 8 weeks of age.

Metabolic Parameters	SS		SS^LepR^mutant	
	Vehicle	Lisinopril	Vehicle	Lisinopril
Body weight (g)	272 ± 16	245 ± 10	365 ± 15^†^	388 ± 13^†^
Glucose (mg/dL)	104 ± 4	96 ± 3	115 ± 8	98 ± 3
Total cholesterol (mg/dL)	175 ± 31	164 ± 15	449 ± 27^†^	314 ± 35^†^^,^^#^

† *indicates a significant difference in p < 0.05 vs. SS rats within the same treatment*.

# *indicates a significant difference in p < 0.05 vs. vehicle-treated rats within the same strain*.

*Values are means ± SE. n = 6–8 per group in each parameter*.

### Measurement of MAP, Proteinuria, and Nephrin Excretion

**Figure 1:**
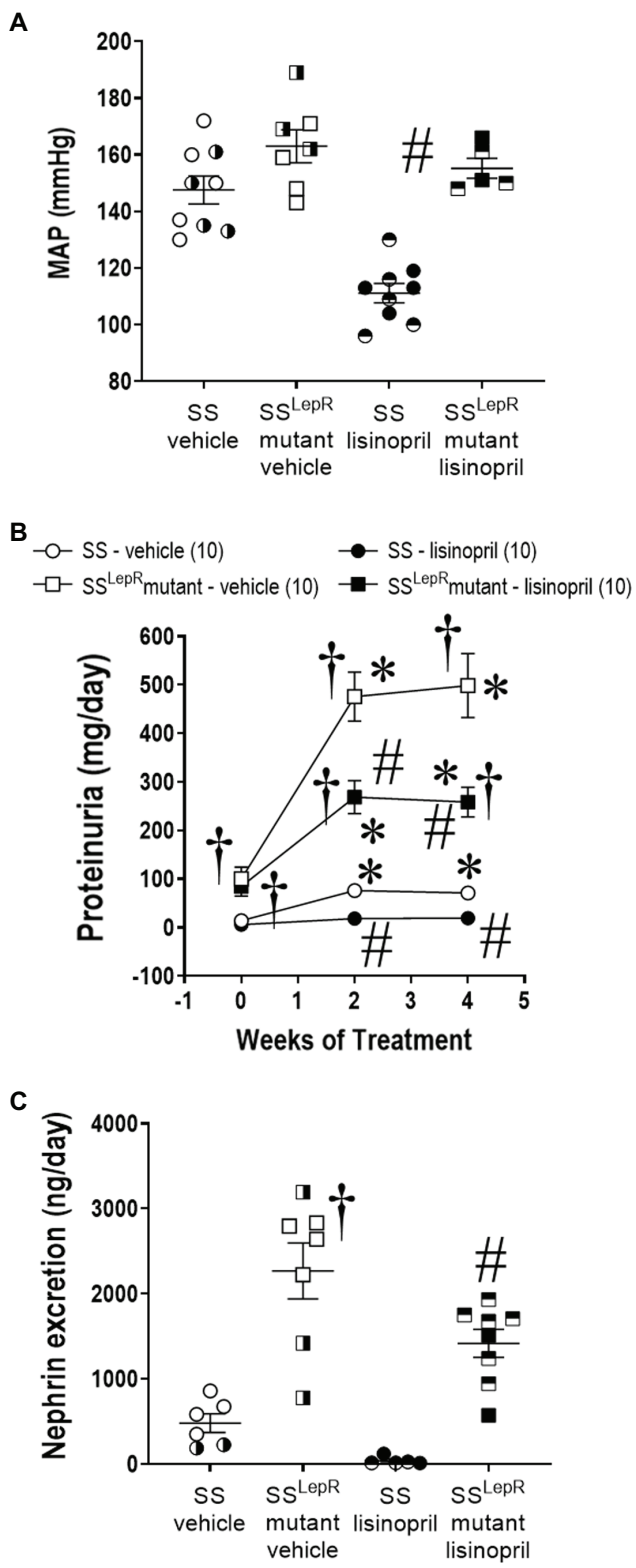
Measurement of mean arterial pressure (MAP) [Panel **(A)**] and temporal changes in proteinuria [Panel **(B)**] and nephrin excretion [Panel **(C)**] in vehicle and lisinopril-treated Dahl salt-sensitive (SS) rats and obese SS leptin receptor mutant (SS^LepR^mutant) rats. Numbers of rats studied (*n* = 5–10 per group). Female rats in each group are represented by partially filled symbols. Values are means ± SE. The significance of the difference in mean values for a single time point was determined by a two-way ANOVA followed by Tukey’s multiple comparisons test. Temporal changes in proteinuria were compared between and within strains using a repeated measures three-way ANOVA followed by the Holm-Sidak test. ^*^indicates a significant difference from the corresponding value within the same strain at baseline, ^†^indicates a significant difference from the corresponding value in SS rats within the same treatment, and ^#^indicates a significant difference from the corresponding value in vehicle within the same strain.

Effects of lisinopril on MAP, proteinuria, and nephrin excretion in SS and SS^LepR^mutant rats are presented in Figure 1. We did not observe a difference in MAP between vehicle-treated SS and SS^LepR^mutant rats (148 ± 5 and 163 ± 6 mmHg, respectively; Figure 1A). Interestingly, lisinopril significantly lowered MAP in SS rats (111 ± 4 mmHg) but not in SS^LepR^mutant rats (155 ± 4 mmHg). At baseline, proteinuria was significantly higher in SS^LepR^mutant rats compared to SS rats (93 ± 22 and 10 ± 4 mg/day, respectively) and remained significantly higher throughout the course of the study (Figure 1B). Chronic treatment with lisinopril significantly reduced proteinuria in SS and SS^LepR^mutant rats compared to their vehicle counterparts (19 ± 5 and 258 ± 34 vs. 71 ± 12 and 498 ± 66 mg/day, respectively). At the end of the study, we measured nephrin excretion to determine podocyte injury (Figure 1C). Nephrin excretion was markedly elevated in vehicle-treated SS^LepR^mutant versus their SS counterparts (2,268 ± 329 vs. 480 ± 110 ng/day, respectively), and treatment with lisinopril significantly reduced nephrin excretion in SS^LepR^mutant rats (1,417 ± 164 ng/day). While treatment with lisinopril reduced nephrin excretion in SS rats (37 ± 21 ng/day), it did not reach statistical significance.

### Measurement of GFR *via* FITC-Sinistrin

**Figure 2:**
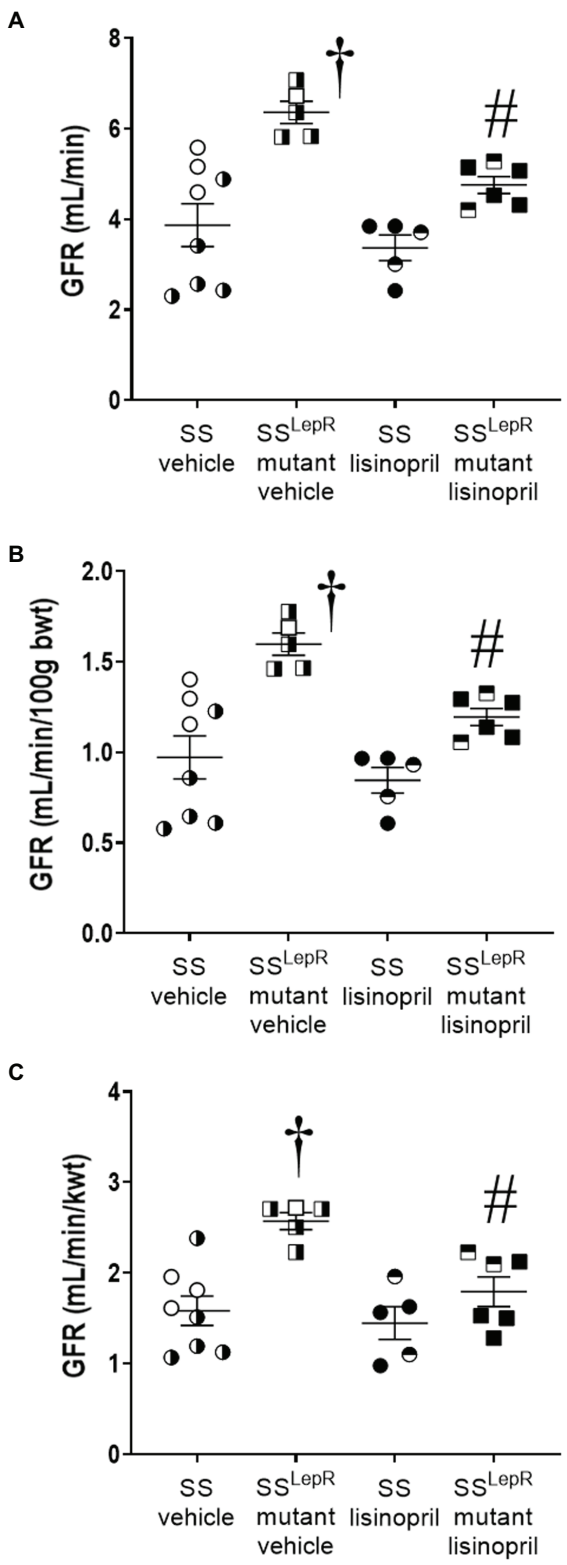
Endpoint measurement of glomerular filtration rate (GFR) by the clearance of FITC-sinistrin not-normalized [Panel **(A)**], normalized to 100 g body weight [Panel **(B)**] and normalized to total kidney weight [Panel **(C)**] in vehicle and lisinopril-treated Dahl salt-sensitive (SS) rats and obese SS^LepR^mutant rats. Numbers of rats studied (*n* = 5–8 per group). Female rats in each group are represented by partially filled symbols. Values are presented as means ± SEM. The significance of the difference in mean values for a single time point was determined by a two-way ANOVA followed by Tukey’s multiple comparisons test. ^†^indicates a significant difference from the corresponding value in SS rats within the same treatment, and ^#^indicates a significant difference from the corresponding value in vehicle within the same strain.

Endpoint measurements of GFR in SS and SS^LepR^mutant rats treated with either vehicle or lisinopril are shown in Figure 2. Unadjusted GFR was 64% higher in SS^LepR^mutant rats compared to SS rats treated with vehicle, and treatment with lisinopril reduced GFR by 30% in SS^LepR^mutant rats without having an effect in SS rats (Figure 2A). When GFR was adjusted to body weight, GFR was significantly higher in vehicle-treated SS^LepR^mutant rats compared to their vehicle-treated SS counterparts (Figure 2B). After 4 weeks of lisinopril treatment, GFR was significantly decreased in SS^LepR^mutant rats compared to the values measured vehicle SS^LepR^mutant rats. Lisinopril treatment had no effect on GFR in SS rats. When adjusting GFR for kidney weight instead body weight, we observed similar results (Figure 2C).

### Glomerular Injury

**Figure 3:**
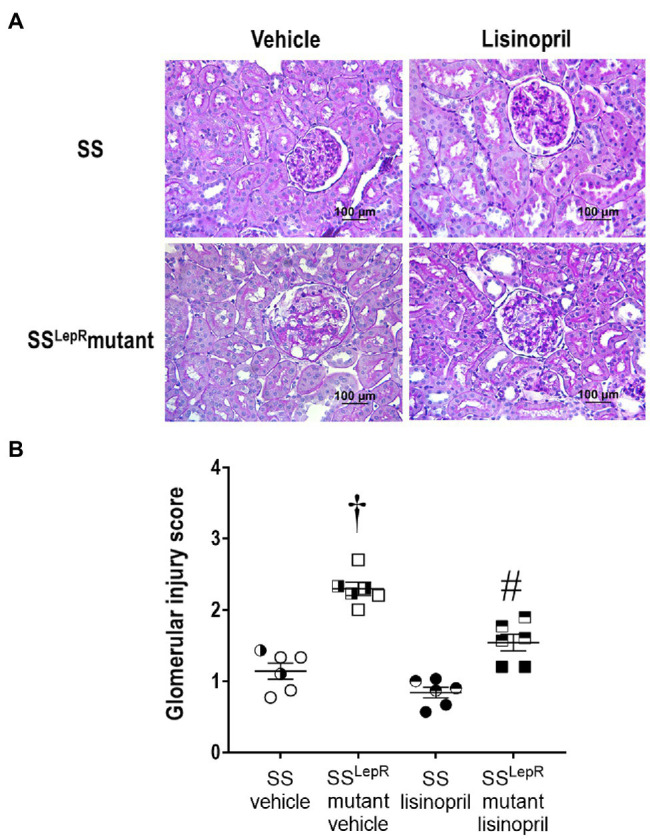
Representative images of renal histopathology: comparison of Periodic acid-Schiff staining [Panel **(A)**] and glomerular injury [Panel **(B)**] in vehicle and lisinopril-treated Dahl salt-sensitive (SS) rats and obese SS^LepR^mutant rats. Numbers of rats studied (*n* = 6 per group). Female rats in each group are represented by partially filled symbols. Values are presented as means ± SEM. The significance of the difference in mean values for a single time point was determined by a two-way ANOVA followed by Tukey’s multiple comparisons test. ^†^indicates a significant difference from the corresponding value in SS rats within the same treatment, and ^#^indicates a significant difference from the corresponding value in vehicle within the same strain.

The effects of treatment with lisinopril on the degree of glomerular injury in SS and SS^LepR^mutant rats are presented in Figure 3. In vehicle-treated animals, SS^LepR^mutant rats displayed a higher degree of mesangial expansion (Figure 3A) and glomerular injury scoring (Figure 3B) when compared to SS rats. Chronic treatment with lisinopril significantly reduced glomerular mesangial expansion and injury in SS^LepR^mutant rats without having an effect in SS rats.

### Comparison of Renal Inflammatory Cytokine Levels

**Figure 4:**
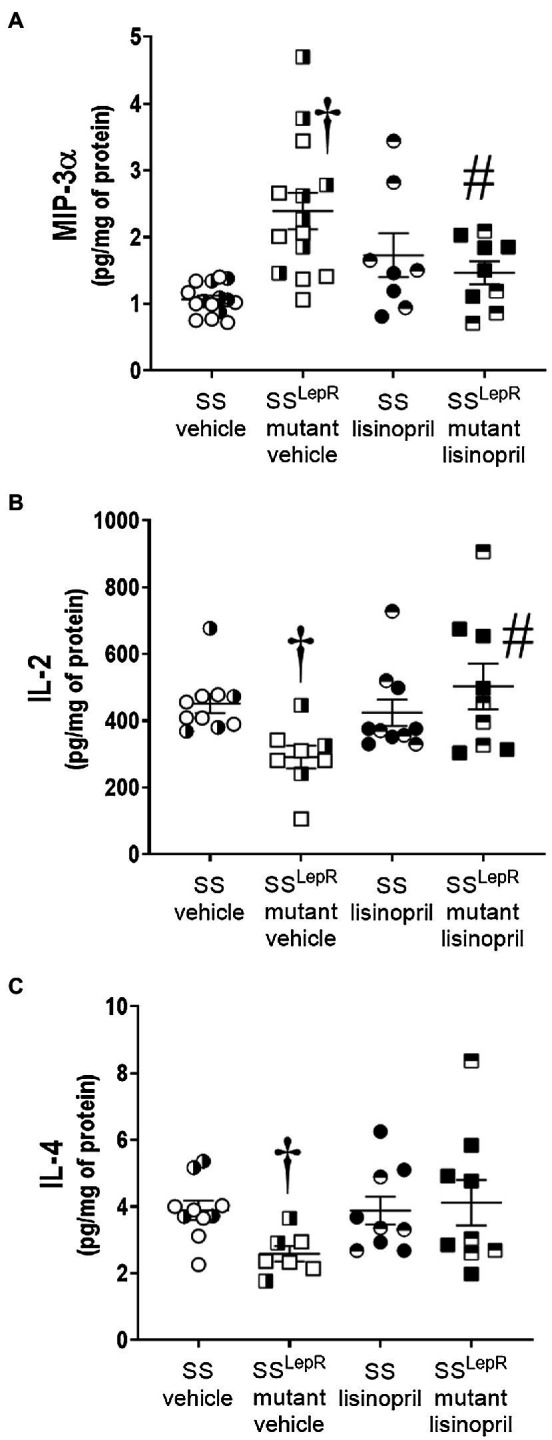
Comparison of renal cytokines levels in vehicle and lisinopril-treated Dahl salt-sensitive (SS) rats and obese SS^LepR^mutant rats: macrophage inflammatory protein-3 alpha [Panel **(A)**], interleukin-2 [Panel **(B)**], and interleukin-4 [Panel **(C)**]. Numbers of rats studied (*n* = 5–8 per group). Female rats in each group are represented by partially filled symbols. Values are presented as means ± SEM. The significance of the difference in mean values for a single time point was determined by a two-way ANOVA followed by Tukey’s multiple comparisons test. ^†^indicates a significant difference from the corresponding value in SS rats within the same treatment, and ^#^indicates a significant difference from the corresponding value in vehicle within the same strain.

The effects of lisinopril on the renal cytokines levels in SS and SS^LepR^mutant rats are presented in Figure 4. Macrophage inflammatory protein-3 alpha (MIP-3α) was increased by more than 2-fold in the kidneys from vehicle-treated SS^LepR^mutant rats compared to SS rats, and chronic treatment with lisinopril significantly reduced renal MIP-3α levels in SS^LepR^mutant rats (Figure 4A). We observed a significant decrease in renal interleukin-2 (IL-2) levels in vehicle-treated SS^LepR^mutant rats compared to the values measured in SS rats, and lisinopril treatment normalized IL-2 levels in the kidneys of SS^LepR^mutant rats (Figure 4B). Similar to IL-2, renal IL-4 levels were decreased by 25% in vehicle-treated SS^LepR^mutant rats versus SS rats, and lisinopril prevented the decrease in IL-4 in SS^LepR^mutant rats (Figure 4C).

## Discussion

Patients suffering from obesity have an increased risk to develop CKD (Chagnac et al., 2000; Bosma et al., 2004; Ejerblad et al., 2006; Hsu et al., 2006; Jacobs et al., 2010). Both childhood and adult obesity are associated with proteinuria and albuminuria (Chagnac et al., 2000; Bosma et al., 2004; Burgert et al., 2006; Ejerblad et al., 2006; Hsu et al., 2006; Jacobs et al., 2010). With childhood obesity increasing at an alarming rate there is a growing need to understand the underlying mechanisms involved. The early stage of renal disease in obese subjects is associated with elevations in GFR or renal hyperfiltration (Ribstein et al., 1995; Chagnac et al., 2000; Zhang and Reisin, 2000; Price et al., 2002; Griffin et al., 2008; McPherson et al., 2020). These functional changes in renal hemodynamics lead to increased transmission of systemic pressure to the glomerulus, which causes damage to the glomerular filtration barrier leading to proteinuria. Previous studies have demonstrated that lowering arterial pressure and GFR attenuates proteinuria, indicating that development of obesity-related proteinuria is due, in part, to alterations in renal hemodynamics (Praga et al., 1999; Chagnac et al., 2000; Bosma et al., 2004; McPherson et al., 2020). These studies suggest that functional changes in the kidney occur in response to weight gain or obesity. Recently, we observed elevations in GFR and glomerular injury in the absence of diabetes and elevations in arterial pressure in obese SS^LepR^mutant rats prior to puberty (McPherson et al., 2016, 2020; Poudel et al., 2018, 2020; Ekperikpe et al., 2021; Shields et al., 2021). Therefore, the current study examined whether treatment with lisinopril to reduce GFR decreases renal inflammation and slows the early progression of proteinuria in SS^LepR^mutant rats. Arterial pressure was similar between SS and SS^LepR^mutant rats treated with vehicle. Interestingly, lisinopril treatment only reduced arterial pressure in SS rats. Chronic treatment with lisinopril significantly reduced proteinuria in SS and SS^LepR^mutant rats compared to their vehicle counterparts. Similar results were observed in nephrin excretion as seen in proteinuria. GFR was significantly elevated in SS^LepR^mutant rats compared to SS rats, and lisinopril treatment reduced GFR by 30% in SS^LepR^mutant rats. The kidneys from SS^LepR^mutant rats displayed glomerular injury and inflammation versus SS rats. Treatment with lisinopril significantly decreased glomerular injury and renal inflammation in the SS^LepR^mutant rats. Overall, these data indicate that inhibiting renal hyperfiltration with an ACE inhibitor is advantageous in preventing the early development of glomerular injury and renal inflammation associated with obesity.

In individuals with and without hyperglycemia, proteinuria is an indicator of future decline in renal function, augmented atherosclerosis, and increased cardiovascular morbidity and mortality (Mogensen, 1984; Gerstein et al., 2001; Hillege et al., 2002). Moreover, patients and animals with high levels of proteinuria display a secondary form of dyslipidemia (Jones et al., 1989; Trevisan et al., 1992; Bruno et al., 1996). Therapies that reduce proteinuria also significantly decrease plasma lipid levels (Gansevoort et al., 1994, 1995; Wapstra et al., 1996; Navis et al., 1997; Buter et al., 2000; Ruggenenti et al., 2003). In the current study, SS^LepR^mutant rats display progressive proteinuria and a three-fold increase in total cholesterol levels compared to their SS counterparts, and chronic treatment with lisinopril significantly reduced both, proteinuria and total cholesterol levels. The lipid-lowering effect of lisinopril could be considered a rare observation, since ACE inhibition does not have a direct impact on plasma lipid levels. However, previous studies have demonstrated therapies, such as ACE inhibitors, which decrease proteinuria, reduce plasma lipid levels as well. There are two possible hypotheses by which progressive proteinuria contributes to dyslipidemia. One hypothesis is that proteinuria causes reduced plasma albumin levels by stimulating liver-derived albumin synthesis, which in turn also involves the synthesis and secretion of other lipoproteins and lipids into the circulation (Marsh and Drabkin, 1960; Jones et al., 1967; Vaziri et al., 2001, 2003). This hypothesis would explain the increased lipogenesis observed during progressive proteinuria but does not account for the removal of these elevated lipids. The other hypothesis involves the urinary loss of heparin sulfate, which is a cofactor for the liporegulator, lipoprotein lipase, and ultimately causes defective removal of lipids from the circulation. In support of this hypothesis, patients with severe proteinuria and renal injury have high levels of heparin sulfate lost in the urine (Staprans et al., 1980, 1987; Vaziri, 2003). These data suggest that chronic ACE inhibition provides beneficial effects, such as lowering lipid levels secondary to reducing proteinuria during the early progression of renal injury associated with obesity.

Another major finding in the current study is that chronic treatment with lisinopril significantly reduced arterial pressure in lean SS rats but not in the obese SS^LepR^mutant rats. Although ACE inhibitors have been shown to effectively reduce arterial pressure in both hypertensive and normotensive patients, they have proven to have a greater efficacy in decreasing arterial pressure in patients with the highest levels of plasma renin activity (Vidt et al., 1982; Todd and Heel, 1986; Pool et al., 1987). Previous studies have demonstrated that SS rats are considered a low renin model of hypertension and are resistant to ACE inhibitors (Jama et al., 2021). However, there are three possible explanations for this interesting finding. (Chagnac et al., 2000) While we did not measure food intake in the current study, leptin signaling deficient models of obesity eat more food than their lean control counterparts (Fantuzzi and Faggioni, 2000; Zhang and Reisin, 2000). This would lead to a higher sodium intake in the SS^LepR^mutant rats contributing to a lower plasma renin activity compared to SS rats, therefore explaining the contrasting effects of ACE inhibition on arterial pressure between the two strains. Moreover, the rats in the current study were not fed your typical high salt diet (diet containing 1% NaCl vs. diets containing 4–8% NaCl). To our knowledge, this is one of the first studies examining the effects of an ACE inhibitor on cardiorenal disease in obese SS rats. (Ejerblad et al., 2006) Another possible reason for the lack of arterial pressure reduction to an ACE inhibitor in the SS^LepR^mutant strain is the impact of other obesity-related mediators (i.e., endothelin, 20-HETE, and catecholamines) that could contribute to the maintenance of arterial pressure. (Jacobs et al., 2010) The third potential explanation is the age at which the rats were treated with an ACE inhibitor. Previous studies have reported that ACE inhibitors reduce MAP in older age obese rats with proteinuria (González-Albarrán et al., 2003; Toblli et al., 2004; Moulana and Maranon, 2018). In the current study, treatment with lisinopril occurred prior to puberty (≤ 8 weeks of age), which may explain the lack of an arterial pressure-lowering effect in response to ACE inhibition in SS^LepR^mutant rats. These results suggest that the arterial pressure-lowering effects of ACE inhibitors in obese individuals and animals may be salt- and age-sensitive, and further studies are needed to investigate these effects in obese young children.

ACE inhibitors are considered one of the standard treatments for obese and diabetic patients with renal disease (Agodoa et al., 2001; Jafar et al., 2001; Progression of Chronic Kidney Disease, 2003; Chu et al., 2021). During the early stages of obesity and diabetes-induced renal disease, constriction of the efferent arteriole of the glomerulus by AngII contributes to elevations in GFR (Hall, 1986, 1991). We hypothesize that the elevations in GFR trigger the early development of glomerular injury and proteinuria in obese and diabetic individuals. Treatment with ACE inhibitors block the formation of AngII and causes vasodilation of the efferent renal artery, which results in a decrease in GFR (Navis et al., 1996). In the current study, GFR was increased by 30% in SS^LepR^mutant rats compared to SS rats and preventing the elevation in GFR with the ACE inhibitor, lisinopril, markedly decreased glomerular injury and proteinuria. Similar results were observed in studies performed by Kojima et al., in which lisinopril deceased arterial pressure and GFR and attenuated the progression of proteinuria in diabetic SS and type-2 diabetic nephropathy rats (Kojima et al., 2013, 2015). However, the beneficial effects of lisinopril observed in SS^LepR^mutant rats were independent of reducing arterial pressure. These data suggest the early progression of proteinuria in SS^LepR^mutant rats is attributed to elevations in GFR and the chronic treatment with lisinopril is effective in reducing proteinuria without affecting arterial pressure.

Similar to our previous studies SS^LepR^mutant rats developed progressive glomerular injury and proteinuria compared to SS rats prior to puberty (<8 weeks of age) (McPherson et al., 2016, 2020; Poudel et al., 2018, 2020; Ekperikpe et al., 2021; Shields et al., 2021). The early progression of proteinuria in SS^LepR^mutant rats is associated with elevations in GFR and renal inflammation (McPherson et al., 2020; Poudel et al., 2020). Therefore, the current study examined the effects ACE inhibition on proteinuria and glomerular injury during the prepubescent stage in SS^LepR^mutant rats. Since ACE inhibition had such a profound effect on GFR, the decrease in glomerular injury and proteinuria in SS^LepR^mutant rats was not surprising. Moreover, renal disease is associated with increased inflammation and pro-inflammatory cytokines (Bemelmans et al., 1993; Himmelfarb et al., 2002; Glorieux et al., 2004; Garibotto et al., 2007; Carrero et al., 2009; Massy et al., 2009; Gosmanova and Le, 2011; Schepers et al., 2011; Gupta et al., 2012; Adesso et al., 2013). Inflammation amplifies renal damage contributing to both acute kidney injury and CKD and has been suggested as a potential therapeutic target for the treatment of renal injury (Bonventre and Yang, 2011; Meng et al., 2014; Jang and Rabb, 2015). When stressed or injured, numerous cells types in the kidney (endothelial cells, podocytes, mesangial cells, tubular epithelial cells, and interstitial fibroblasts) produce inflammatory mediators (i.e., chemokines and cytokines) and stimulate an immune response (Woltman et al., 2005; Villa et al., 2013; Lu et al., 2017; Zhao et al., 2017; Srivastava et al., 2018). In the current study, the pro-inflammatory chemokine, MIP3-α, was significantly increased in the kidneys from SS^LepR^mutant rats compared to SS rats. Turner et al. demonstrated that increasing MIP3-α stimulates immune cell recruitment, renal injury, albuminuria, and reduced renal function in a mouse model of nephrotoxic nephritis (Turner et al., 2010). Chronic treatment with lisinopril decreased MIP3-α in the kidneys from SS^LepR^mutant rats. Moreover, we previously reported that chronic blockade of MIP3-α reduced renal injury in SS^LepR^mutant rats (Ekperikpe et al., 2021). Renal IL-2 and IL-4 were significantly decreased in SS^LepR^mutant rats versus SS rats, and both have been shown to play a major role in immune cell differentiation and homeostasis (Abbas et al., 2018; Junttila, 2018; Ross and Cantrell, 2018; Kassem et al., 2020). We observed that lisinopril prevented the decrease in the renal levels of IL-2 and IL-4. How these cytokines influence the early progression of glomerular injury during obesity remains to be determined. Similarly, we also observed that chronic treatment with lisinopril reduced proteinuria in lean SS rats, which is more than likely due to the arterial pressure-lowering effect of ACE inhibition in this strain. However, lisinopril did not reduce inflammation in SS rats, but the degree of glomerular injury and proteinuria in SS rats was not as severe as observed in SS^LepR^mutant rats, which may explain the differences between the two strains. Overall, these results suggest that ACE inhibition reduces the early development of glomerular injury by not only reducing GFR but also *via* decreasing renal inflammation.

### Clinical Translational Perspective

With childhood obesity on rise and obese children displaying early signs of proteinuria and hypertension, there is an important need to study the mechanisms involved in the early development of proteinuria-associated obesity. Although dysfunctional leptin signaling obese models are not the ideal model to study obesity renal disease, SS^LepR^mutant rats display progressive proteinuria and glomerular injury that is associated with renal hyperfiltration prior to puberty (McPherson et al., 2020). The current study examined whether renal hyperfiltration plays important role in the early progression of proteinuria in SS^LepR^mutant rats by using the ACE inhibitor, lisinopril. ACE inhibitor therapy is a common method of treatment for obese individuals with proteinuria (Agodoa et al., 2001; Jafar et al., 2001; Progression of Chronic Kidney Disease, 2003; Chu et al., 2021). We observed that chronic treatment with lisinopril prevented renal hyperfiltration and reduced glomerular injury, proteinuria, and renal inflammation in SS^LepR^mutant rats independent of lowering arterial pressure. To our knowledge, the current study is one of the first studies to examine effects of ACE inhibition on the early changes in GFR and progression of renal disease in an obese animal model during the prepubescent stage. Moreover, further studies are needed to investigate the alterations in GFR and renal disease and the impact of ACE inhibitors on renal hemodynamics within this young obese population.

## Data Availability Statement

The raw data supporting the conclusions of this article will be made available by the authors, without undue reservation.

## Ethics Statement

The animal study was reviewed and approved by University of Mississippi Medical Center Institutional Animal Care and Use Committee.

## Author Contributions

AB, AN, and JW provided conception and prepared the figures. AB, AN, UE, KM, CS, BP, CC, DC, and JW drafted, edited, and revised the manuscript and approved the final version of the manuscript. All authors contributed to the article and approved the submitted version.

## Funding

This work was financially supported by the National Institutes of Diabetes and Digestive and Kidney Diseases of the National Institutes of Health (DK109133) awarded to JW and the National Heart, Lung and Blood Institute of the National Institutes of Health T32 Grant (T32HL105324) awarded to AB and (HL151407) awarded to DC. The work performed through the UMMC Molecular and Genomics Facility was supported, in part, by funds from the NIGMS, including the Mississippi INBRE (P20GM103476), Obesity, Cardiorenal and Metabolic Diseases-COBRE (P20GM104357), and the Mississippi Center of Excellence in Perinatal Research (MS-CEPR)-COBRE (P20GM121334). The content was solely the responsibility of the authors and does not necessarily represent the official views of the National Institutes of Health.

## Conflict of Interest

The authors declare that the research was conducted in the absence of any commercial or financial relationships that could be construed as a potential conflict of interest.

## Publisher’s Note

All claims expressed in this article are solely those of the authors and do not necessarily represent those of their affiliated organizations, or those of the publisher, the editors and the reviewers. Any product that may be evaluated in this article, or claim that may be made by its manufacturer, is not guaranteed or endorsed by the publisher.

## References

[ref1] Abbas A. K. Trotta E. R. Simeonov D. Marson A. Bluestone J. A. (2018). Revisiting IL-2: biology and therapeutic prospects. Science Immunol. 3:eaat1482. doi: 10.1126/sciimmunol.aat1482, PMID: 29980618

[ref2] Adesso S. Popolo A. Bianco G. Sorrentino R. Pinto A. Autore G.. (2013). The uremic toxin indoxyl sulphate enhances macrophage response to LPS. PLoS One 8:e76778. doi: 10.1371/journal.pone.0076778, PMID: 24098806 PMC3786936

[ref3] Agodoa L. Y. Appel L. Bakris G. L. Beck G. Bourgoignie J. Briggs J. P.. (2001). Effect of Ramipril vs amlodipine on renal outcomes in hypertensive NephrosclerosisA randomized controlled trial. JAMA 285, 2719–2728. doi: 10.1001/jama.285.21.2719, PMID: 11386927

[ref4] Alique M. Civantos E. Sanchez-Lopez E. Lavoz C. Rayego-Mateos S. Rodrigues-Díez R.. (2014). Integrin-linked kinase plays a key role in the regulation of angiotensin II-induced renal inflammation. Clin. Sci. 127, 19–31. doi: 10.1042/CS20130412, PMID: 24383472

[ref5] Bemelmans M. H. Gouma D. J. Buurman W. A. (1993). Influence of nephrectomy on tumor necrosis factor clearance in a murine model. J. Immunol. 150, 2007–2017. 8436831

[ref6] Bonventre J. V. Yang L. (2011). Cellular pathophysiology of ischemic acute kidney injury. J. Clin. Invest. 121, 4210–4221. doi: 10.1172/JCI45161, PMID: 22045571 PMC3204829

[ref7] Bosma R. J. van der Heide J. J. Oosterop E. J. de Jong P. E. Navis G. (2004). Body mass index is associated with altered renal hemodynamics in non-obese healthy subjects. Kidney Int. 65, 259–265. doi: 10.1111/j.1523-1755.2004.00351.x, PMID: 14675058

[ref8] Bruno G. Cavallo-Perin P. Bargero G. Borra M. Calvi V. D’Errico N.. (1996). Prevalence and risk factors for micro- and macroalbuminuria in an Italian population-based cohort of NIDDM subjects. Diabetes Care 19, 43–47. doi: 10.2337/diacare.19.1.43, PMID: 8720532

[ref9] Burgert T. S. Dziura J. Yeckel C. Taksali S. E. Weiss R. Tamborlane W.. (2006). Microalbuminuria in pediatric obesity: prevalence and relation to other cardiovascular risk factors. Int. J. Obes. 30, 273–280. doi: 10.1038/sj.ijo.0803136, PMID: 16231019

[ref10] Buter H. van Tol A. Navis G. J. Scheek L. M. de Jong P. E. de Zeeuw D.. (2000). Angiotensin II receptor antagonist treatment lowers plasma total and very low + low density lipoprotein cholesterol in type 1 diabetic patients with albuminuria without affecting plasma cholesterol esterification and cholesteryl ester transfer. Diabet. Med. 17, 550–552. doi: 10.1046/j.1464-5491.2000.00311.x, PMID: 10972588

[ref11] Carrero J. J. Park S. H. Axelsson J. Lindholm B. Stenvinkel P. (2009). Cytokines, atherogenesis, and hypercatabolism in chronic kidney disease: a dreadful triad. Semin. Dial. 22, 381–386. doi: 10.1111/j.1525-139X.2009.00585.x, PMID: 19708986

[ref12] Chagnac A. Weinstein T. Korzets A. Ramadan E. Hirsch J. Gafter U. (2000). Glomerular hemodynamics in severe obesity. Am. J. Physiol. Renal Physiol. 278, F817–F822. doi: 10.1152/ajprenal.2000.278.5.F817, PMID: 10807594

[ref13] Chagnac A. Weinstein T. Korzets A. Ramadan E. Hirsch J. Gafter U. (2000). Glomerular hemodynamics in severe obesity. American journal of physiology-renal. Physiology 278, F817–F822. doi: 10.1152/ajprenal.2000.278.5.F817 10807594

[ref14] Chen J. Muntner P. Hamm L. L. Jones D. W. Batuman V. Fonseca V.. (2004). The metabolic syndrome and chronic kidney disease in U.S. adults. Ann. Intern. Med. 140, 167–174. doi: 10.7326/0003-4819-140-3-200402030-00007, PMID: 14757614

[ref15] Chu C. D. Powe N. R. McCulloch C. E. Banerjee T. Crews D. C. Saran R.. (2021). Angiotensin-converting enzyme inhibitor or angiotensin receptor blocker use Among hypertensive US adults With albuminuria. Hypertension 77, 94–102. doi: 10.1161/HYPERTENSIONAHA.120.16281, PMID: 33190561 PMC7725867

[ref16] Ejerblad E. Fored C. M. Lindblad P. Fryzek J. McLaughlin J. K. Nyren O. (2006). Obesity and risk for chronic renal failure. J Am Soc Nephrol 17, 1695–1702. doi: 10.1681/ASN.2005060638, PMID: 16641153

[ref17] Ekperikpe U. Poudel B. Shields C. Brown A. Cornelius D. Williams J. (2021). Administration of MIP3-alpha neutralizing antibody reduces the renal infiltration of dendritic cells and Th17s and attenuates progressive proteinuria in obese dahl salt-sensitive rats. FASEB J. 35. doi: 10.1096/fasebj.2021.35.S1.02451

[ref18] Fantuzzi G. Faggioni R. (2000). Leptin in the regulation of immunity, inflammation, and hematopoiesis. J. Leukoc. Biol. 68, 437–446. PMID: 11037963

[ref19] Gansevoort R. T. de Zeeuw D. de Jong P. E. (1995). Additive antiproteinuric effect of ACE inhibition and a low-protein diet in human renal disease. Nephrol. Dial. Transplant. 10, 497–504. doi: 10.1093/ndt/10.4.497, PMID: 7623991

[ref20] Gansevoort R. T. Heeg J. E. Dikkeschei F. D. de Zeeuw D. de Jong P. E. Dullaart R. P. (1994). Symptomatic antiproteinuric treatment decreases serum lipoprotein (a) concentration in patients with glomerular proteinuria. Nephrol. Dial. Transplant. 9, 244–250. PMID: 8052429

[ref21] Garibotto G. Sofia A. Balbi M. Procopio V. Villaggio B. Tarroni A.. (2007). Kidney and splanchnic handling of interleukin-6 in humans. Cytokine 37, 51–54. doi: 10.1016/j.cyto.2007.02.015, PMID: 17420140

[ref22] Gerstein H. C. Mann J. F. Yi Q. Zinman B. Dinneen S. F. Hoogwerf B.. (2001). Albuminuria and risk of cardiovascular events, death, and heart failure in diabetic and nondiabetic individuals. JAMA 286, 421–426. doi: 10.1001/jama.286.4.421, PMID: 11466120

[ref23] Glorieux G. L. Dhondt A. W. Jacobs P. Van Langeraert J. Lameire N. H. De Deyn P. P.. (2004). In vitro study of the potential role of guanidines in leukocyte functions related to atherogenesis and infection. Kidney Int. 65, 2184–2192. doi: 10.1111/j.1523-1755.2004.00631.x, PMID: 15149331

[ref24] González-Albarrán O. Gómez O. Ruiz E. Vieitez P. García-Robles R. (2003). Role of systolic blood pressure on the progression of kidney damage in an experimental model of type 2 diabetes mellitus, obesity, and hypertension (Zucker rats). Am. J. Hypertens. 16, 979–985. doi: 10.1016/S0895-7061(03)01000-8 14573338

[ref25] Gosmanova E. O. Le N. A. (2011). Cardiovascular complications in CKD patients: role of oxidative stress. Cardiol. Res. Pract. 2011:156326. doi: 10.4061/2011/156326, PMID: 21253517 PMC3022166

[ref26] Griffin K. A. Kramer H. Bidani A. K. (2008). Adverse renal consequences of obesity. American journal of physiology-renal. Physiology 294, F685–F696. doi: 10.1152/ajprenal.00324.2007 18234955

[ref27] Gupta J. Mitra N. Kanetsky P. A. Devaney J. Wing M. R. Reilly M.. (2012). Association between albuminuria, kidney function, and inflammatory biomarker profile in CKD in CRIC. CJASN 7, 1938–1946. doi: 10.2215/CJN.03500412, PMID: 23024164 PMC3513744

[ref28] Hales C. M. Carroll M. D. Fryar C. D. Ogden C. L. (2017). Prevalence of obesity Among adults and youth: United States, 2015-2016. NCHS Data Brief 288, 1–8, PMID: . 29155689

[ref29] Hall J. E. (1986). Regulation of glomerular filtration rate and sodium excretion by angiotensin II. Fed. Proc. 45, 1431–1437. PMID: 3514280

[ref30] Hall J. E. (1991). The renin-angiotensin system: renal actions and blood pressure regulation. Compr. Ther. 17, 8–17. PMID: 1879129

[ref31] Hall J. E. Henegar J. R. Dwyer T. M. Liu J. Da Silva A. A. Kuo J. J.. (2004). Is obesity a major cause of chronic kidney disease? Adv. Ren. Replace. Ther. 11, 41–54. doi: 10.1053/j.arrt.2003.10.007, PMID: 14730537

[ref32] Henegar J. R. Bigler S. A. Henegar L. K. Tyagi S. C. Hall J. E. (2001). Functional and structural changes in the kidney in the early stages of obesity. J. Am. Soc. Nephrol. 12, 1211–1217. doi: 10.1681/ASN.V1261211, PMID: 11373344

[ref33] Hillege H. L. Fidler V. Diercks G. F. van Gilst W. H. de Zeeuw D. van Veldhuisen D. J.. (2002). Urinary albumin excretion predicts cardiovascular and noncardiovascular mortality in general population. Circulation 106, 1777–1782. doi: 10.1161/01.CIR.0000031732.78052.81, PMID: 12356629

[ref34] Himmelfarb J. Stenvinkel P. Ikizler T. A. Hakim R. M. (2002). The elephant in uremia: oxidant stress as a unifying concept of cardiovascular disease in uremia. Kidney Int. 62, 1524–1538. doi: 10.1046/j.1523-1755.2002.00600.x, PMID: 12371953

[ref35] Hsu C. Y. McCulloch C. E. Iribarren C. Darbinian J. Go A. S. (2006). Body mass index and risk for end-stage renal disease. Ann. Intern. Med. 144, 21–28. doi: 10.7326/0003-4819-144-1-200601030-00006, PMID: 16389251

[ref36] Jacobs E. J. Newton C. C. Wang Y. Patel A. V. McCullough M. L. Campbell P. T.. (2010). Waist circumference and all-cause mortality in a large US cohort. Arch. Intern. Med. 170, 1293–1301. doi: 10.1001/archinternmed.2010.201, PMID: 20696950

[ref37] Jafar T. H. Schmid C. H. Landa M. Giatras I. Toto R. Remuzzi G.. (2001). Angiotensin-converting enzyme inhibitors and progression of nondiabetic renal disease. Ann. Intern. Med. 135, 73–87. doi: 10.7326/0003-4819-135-2-200107170-00007 11453706

[ref38] Jama H. A. R. Muralitharan R. Xu C. O’Donnell J. Bertagnolli M. Broughton B.. (2021). “Rodent models of hypertension,” *British Journal of Pharmacology*; August 7, 2021. 10.1111/bph.15650 34363610

[ref39] Jang H. R. Rabb H. (2015). Immune cells in experimental acute kidney injury. Nat. Rev. Nephrol. 11, 88–101. doi: 10.1038/nrneph.2014.180, PMID: 25331787

[ref40] Jones S. L. Close C. F. Mattock M. B. Jarrett R. J. Keen H. Viberti G. C. (1989). Plasma lipid and coagulation factor concentrations in insulin dependent diabetics with microalbuminuria. BMJ 298, 487–490. doi: 10.1136/bmj.298.6672.487, PMID: 2495077 PMC1835810

[ref41] Jones A. L. Ruderman N. B. Herrera M. G. (1967). Electron microscopic and biochemical study of lipoprotein synthesis in the isolated perfused rat liver. J. Lipid Res. 8, 429–446. doi: 10.1016/S0022-2275(20)38900-8, PMID: 6049670

[ref42] Junttila I. S. (2018). Tuning the cytokine responses: An update on interleukin (IL)-4 and IL-13 receptor complexes. Front. Immunol. 9:888. doi: 10.3389/fimmu.2018.00888 29930549 PMC6001902

[ref43] Kalupahana N. S. Moustaid-Moussa N. (2012). The renin-angiotensin system: a link between obesity, inflammation and insulin resistance. Obes. Rev. 13, 136–149. doi: 10.1111/j.1467-789X.2011.00942.x, PMID: 22034852

[ref44] Kalupahana N. S. Moustaid-Moussa N. Claycombe K. J. (2012). Immunity as a link between obesity and insulin resistance. Mol. Asp. Med. 33, 26–34. doi: 10.1016/j.mam.2011.10.011, PMID: 22040698

[ref45] Kassem K. M. Ali M. Rhaleb N.-E. (2020). Interleukin 4: its role in hypertension, atherosclerosis, Valvular, and Nonvalvular cardiovascular diseases. J. Cardiovasc. Pharmacol. Ther. 25, 7–14. doi: 10.1177/1074248419868699, PMID: 31401864 PMC6904928

[ref46] Kojima N. Williams J. M. Slaughter T. N. Kato S. Takahashi T. Miyata N.. (2015). Renoprotective effects of combined SGLT 2 and ACE inhibitor therapy in diabetic D ahl S rats. Physiol. Rep. 3:e12436. doi: 10.14814/phy2.12436, PMID: 26169541 PMC4552522

[ref47] Kojima N. Williams J. M. Takahashi T. Miyata N. Roman R. J. (2013). Effects of a new SGLT2 inhibitor, luseogliflozin, on diabetic nephropathy in T2DN rats. J. Pharmacol. Exp. Ther. 345, 464–472. doi: 10.1124/jpet.113.203869, PMID: 23492941 PMC3657104

[ref48] Kurella M. Lo J. C. Chertow G. M. (2005). Metabolic syndrome and the risk for chronic kidney disease among nondiabetic adults. J Am Soc Nephrol 16, 2134–2140. doi: 10.1681/ASN.2005010106, PMID: 15901764

[ref49] Li C. Culver S. A. Quadri S. Ledford K. L. Al-Share Q. Y. Ghadieh H. E.. (2015). High-fat diet amplifies renal renin angiotensin system expression, blood pressure elevation, and renal dysfunction caused by Ceacam1 null deletion. Am. J. Physiol. Endocrinol. Metab. 309, E802–E810. doi: 10.1152/ajpendo.00158.2015, PMID: 26374765 PMC4628940

[ref50] Lu G. Zhang X. Shen L. Qiao Q. Li Y. Sun J.. (2017). CCL20 secreted from IgA1-stimulated human mesangial cells recruits inflammatory Th17 cells in IgA nephropathy. PLoS One 12:e0178352. doi: 10.1371/journal.pone.0178352, PMID: 28552941 PMC5446182

[ref51] Marsh J. B. Drabkin D. L. (1960). Experimental reconstruction of metabolic pattern of lipid nephrosis: key role of hepatic protein synthesis in hyperlipemia. Metabolism 9, 946–955. PMID: 13767173

[ref52] Massy Z. A. Stenvinkel P. Drueke T. B. (2009). The role of oxidative stress in chronic kidney disease. Semin. Dial. 22, 405–408. doi: 10.1111/j.1525-139X.2009.00590.x, PMID: 19708991

[ref53] McPherson K. C. Shields C. A. Poudel B. Johnson A. C. Taylor L. Stubbs C.. (2020). Altered renal hemodynamics is associated with glomerular lipid accumulation in obese dahl salt-sensitive leptin receptor mutant rats. Am. J. Physiol. Renal Physiol. 318, F911–F921. doi: 10.1152/ajprenal.00438.2019, PMID: 32068459 PMC7191445

[ref54] McPherson K. C. Taylor L. Johnson A. C. Didion S. P. Geurts A. M. Garrett M. R.. (2016). Early development of podocyte injury independently of hyperglycemia and elevations in arterial pressure in nondiabetic obese dahl SS leptin receptor mutant rats. Am. J. Physiol. Renal Physiol. 311, F793–F804. doi: 10.1152/ajprenal.00590.2015, PMID: 27465994 PMC5142236

[ref55] Meng X. M. Nikolic-Paterson D. J. Lan H. Y. (2014). Inflammatory processes in renal fibrosis. Nat. Rev. Nephrol. 10, 493–503. doi: 10.1038/nrneph.2014.114, PMID: 24981817

[ref56] Mogensen C. E. (1984). Microalbuminuria predicts clinical proteinuria and early mortality in maturity-onset diabetes. N. Engl. J. Med. 310, 356–360. doi: 10.1056/NEJM198402093100605, PMID: 6690964

[ref57] Moulana M. Maranon R. O. (2018). Regulation of blood pressure is influenced by gender: A study in obese Zucker rats. Life Sci. 209, 236–241. doi: 10.1016/j.lfs.2018.08.020, PMID: 30098343 PMC6639019

[ref58] Navis G. Buter H. de Jong P. E. Dullaart R. P. de Zeeuw D. (1997). Effect of antiproteinuric treatment on the lipid profile in nondiabetic renal disease. Contrib. Nephrol. 120, 88–96. 9257051 10.1159/000059827

[ref59] Navis G. Faber H. J. de Zeeuw D. de Jong P. E. (1996). ACE inhibitors and the kidney. Drug Saf. 15, 200–211. doi: 10.2165/00002018-199615030-00005, PMID: 8879974

[ref60] Ogden C. L. Carroll M. D. Lawman H. G. Fryar C. D. Kruszon-Moran D. Kit B. K.. (2016). Trends in obesity prevalence Among children and adolescents in the United States, 1988-1994 Through 2013-2014. JAMA 315, 2292–2299. doi: 10.1001/jama.2016.6361, PMID: 27272581 PMC6361521

[ref61] Pill J. Issaeva O. Woderer S. Sadick M. Kränzlin B. Fiedler F.. (2006). Pharmacological profile and toxicity of fluorescein-labelled sinistrin, a novel marker for GFR measurements. Naunyn Schmiedeberg’s Arch. Pharmacol. 373, 204–211. doi: 10.1007/s00210-006-0067-0, PMID: 16736157

[ref62] Pill J. Kraenzlin B. Jander J. Sattelkau T. Sadick M. Kloetzer H.-M.. (2005). Fluorescein-labeled sinistrin as marker of glomerular filtration rate. Eur. J. Med. Chem. 40, 1056–1060. doi: 10.1016/j.ejmech.2005.03.020, PMID: 15919135

[ref63] Pool J. L. Gennari J. Goldstein R. Kochar M. S. Lewin A. J. Maxwell M. H.. (1987). Controlled multicenter study of the antihypertensive effects of lisinopril, hydrochlorothiazide, and lisinopril plus hydrochlorothiazide in the treatment of 394 patients with mild to moderate essential hypertension. J. Cardiovasc. Pharmacol. 9, S36–S42. doi: 10.1097/00005344-198700003-00010, PMID: 2442550

[ref64] Poudel B. Shields C. A. Brown A. K. Ekperikpe U. Johnson T. Cornelius D. C.. (2020). Depletion of macrophages slows the early progression of renal injury in obese dahl salt-sensitive leptin receptor mutant rats. Am. J. Physiol. Renal Physiol. 318, F1489–F1499. doi: 10.1152/ajprenal.00100.2020, PMID: 32390513 PMC7311704

[ref65] Poudel B. Shields C. A. Cornelius D. C. Williams J. M. (2018). Sex differences in the development of renal injury in obese dahl salt-sensitive leptin receptor mutant rats During Prepubertal obesity. FASEB J. 32:906-5. doi: 10.1096/fasebj.2018.32.1_supplement.906.529046358

[ref66] Praga M. Morales E. Herrero J. C. Perez Campos A. Dominguez-Gil B. Alegre R.. (1999). Absence of hypoalbuminemia despite massive proteinuria in focal segmental glomerulosclerosis secondary to hyperfiltration. Am. J. Kidney Diseases 33, 52–58. doi: 10.1016/S0272-6386(99)70257-X, PMID: 9915267

[ref67] Price D. A. Lansang M. C. Osei S. Y. Fisher N. D. L. Laffel L. M. B. Hollenberg N. K. (2002). Type 2 diabetes, obesity, and the renal response to blocking the renin system with irbesartan. Diabet. Med. 19, 858–861. doi: 10.1046/j.1464-5491.2002.00806.x, PMID: 12358875

[ref68] Progression of Chronic Kidney Disease (2003). The role of blood pressure control, proteinuria, and angiotensin-converting enzyme inhibition: A patient-level meta-analysis. Ann. Intern. Med. 139, 244–252. doi: 10.7326/0003-4819-139-4-200308190-00006, PMID: 12965979

[ref69] Ribstein J. Cailar G. Mimran A. (1995). Combined renal effects of overweight and hypertension. Hypertension 26, 610–615. doi: 10.1161/01.HYP.26.4.610, PMID: 7558220

[ref70] Ross S. H. Cantrell D. A. (2018). Signaling and function of Interleukin-2 in T lymphocytes. Annu. Rev. Immunol. 36, 411–413. doi: 10.1146/annurev-immunol-042617-053352, PMID: 29677473 PMC6472684

[ref71] Ruggenenti P. Mise N. Pisoni R. Arnoldi F. Pezzotta A. Perna A.. (2003). Diverse effects of increasing lisinopril doses on lipid abnormalities in chronic nephropathies. Circulation 107, 586–592. doi: 10.1161/01.CIR.0000047526.08376.80, PMID: 12566371

[ref72] Schepers E. Barreto D. V. Liabeuf S. Glorieux G. Eloot S. Barreto F. C.. (2011). Symmetric dimethylarginine as a proinflammatory agent in chronic kidney disease. Clin. J. Am. Soc. Nephrol. 6, 2374–2383. doi: 10.2215/CJN.01720211, PMID: 21817129 PMC3359555

[ref73] Schmieder R. E. Hilgers K. F. Schlaich M. P. Schmidt B. M. (2007). Renin-angiotensin system and cardiovascular risk. Lancet 369, 1208–1219. doi: 10.1016/S0140-6736(07)60242-6, PMID: 17416265

[ref74] Schock-Kusch D. Sadick M. Henninger N. Kraenzlin B. Claus G. Kloetzer H.-M.. (2009). Transcutaneous measurement of glomerular filtration rate using FITC-sinistrin in rats. Nephrol. Dialysis Trans. 24, 2997–3001. doi: 10.1093/ndt/gfp225, PMID: 19461009

[ref75] Schock-Kusch D. Xie Q. Shulhevich Y. Hesser J. Stsepankou D. Sadick M.. (2011). Transcutaneous assessment of renal function in conscious rats with a device for measuring FITC-sinistrin disappearance curves. Kidney Int. 79, 1254–1258. doi: 10.1038/ki.2011.31, PMID: 21368744

[ref76] Shields C. Poudel B. Ekperikpe U. Brown A. Smith S. Cornelius D.. (2021). Sex differences in macrophage polarization During the early progression of renal disease in obese dahl salt-sensitive rats prior to puberty. FASEB J. 35. doi: 10.1096/fasebj.2021.35.S1.02057

[ref77] Srivastava T. Dai H. Heruth D. P. Alon U. S. Garola R. E. Zhou J.. (2018). Mechanotransduction signaling in podocytes from fluid flow shear stress. Am. J. Physiol. Renal Physiol. 314, F22–F34. doi: 10.1152/ajprenal.00325.2017, PMID: 28877882 PMC5866353

[ref78] Staprans I. Anderson C. D. Lurz F. W. Felts J. M. (1980). Separation of a lipoprotein lipase cofactor from the alpha 1-acid glycoprotein fraction from the urine of nephrotic patients. Biochim. Biophys. Acta 617, 514–523. 7370292 10.1016/0005-2760(80)90017-x

[ref79] Staprans I. Felts J. M. Couser W. G. (1987). Glycosaminoglycans and chylomicron metabolism in control and nephrotic rats. Metabolism 36, 496–501. doi: 10.1016/0026-0495(87)90050-3, PMID: 3574136

[ref80] Suzuki Y. Ruiz-Ortega M. Lorenzo O. Ruperez M. Esteban V. Egido J. (2003). Inflammation and angiotensin II. Int. J. Biochem. Cell Biol. 35, 881–900. doi: 10.1016/S1357-2725(02)00271-6, PMID: 12676174

[ref81] Toblli J. E. Cao G. DeRosa G. Gennaro F. D. Forcada P. (2004). Angiotensin-converting enzyme inhibition and angiogenesis in myocardium of obese Zucker rats. Am. J. Hypertens. 17, 172–180. doi: 10.1016/j.amjhyper.2003.10.006, PMID: 14751661

[ref82] Todd P. A. Heel R. C. (1986). Enalapril. A review of its pharmacodynamic and pharmacokinetic properties, and therapeutic use in hypertension and congestive heart failure. Drugs 31, 198–248. doi: 10.2165/00003495-198631030-00002, PMID: 3011386

[ref83] Trevisan R. Nosadini R. Fioretto P. Semplicini A. Donadon V. Doria A.. (1992). Clustering of risk factors in hypertensive insulin-dependent diabetics with high sodium-lithium countertransport. Kidney Int. 41, 855–861. doi: 10.1038/ki.1992.131, PMID: 1513108

[ref84] Turner J. E. Paust H. J. Steinmetz O. M. Peters A. Riedel J. H. Erhardt A.. (2010). CCR6 recruits regulatory T cells and Th17 cells to the kidney in glomerulonephritis. J Am Soc Nephrol 21, 974–985. doi: 10.1681/ASN.2009070741, PMID: 20299360 PMC2900961

[ref85] Vaziri N. D. (2003). Molecular mechanisms of lipid disorders in nephrotic syndrome. Kidney Int. 63, 1964–1976. doi: 10.1046/j.1523-1755.2003.00941.x, PMID: 12675893

[ref86] Vaziri N. D. Liang K. Parks J. S. (2001). Acquired lecithin-cholesterol acyltransferase deficiency in nephrotic syndrome. Am. J. Physiol. Renal Physiol. 280:F823. doi: 10.1152/ajprenal.2001.280.5.F823, PMID: 11292624

[ref87] Vaziri N. D. Sato T. Liang K. (2003). Molecular mechanisms of altered cholesterol metabolism in rats with spontaneous focal glomerulosclerosis. Kidney Int. 63, 1756–1763. doi: 10.1046/j.1523-1755.2003.00911.x, PMID: 12675851

[ref88] Vidt D. G. Bravo E. L. Fouad F. M. (1982). Captopril. N. Engl. J. Med. 306, 214–219. 7033784 10.1056/NEJM198201283060405

[ref89] Villa L. Boor P. Konieczny A. Kunter U. van Roeyen C. R. Denecke B.. (2013). Late angiotensin II receptor blockade in progressive rat mesangioproliferative glomerulonephritis: new insights into mechanisms. J. Pathol. 229, 672–684. doi: 10.1002/path.4151, PMID: 23192593

[ref90] Wapstra F. H. Van Goor H. Navis G. De Jong P. E. De Zeeuw D. (1996). Antiproteinuric effect predicts renal protection by angiotensin-converting enzyme inhibition in rats with established adriamycin nephrosis. Clin. Sci. 90, 393–401. doi: 10.1042/cs0900393, PMID: 8665777

[ref91] Woltman A. M. de Fijter J. W. van der Kooij S. W. Jie K. E. Massacrier C. Caux C.. (2005). MIP-3alpha/CCL20 in renal transplantation and its possible involvement as dendritic cell chemoattractant in allograft rejection. Am. J. Transplant. 5, 2114–2125. doi: 10.1111/j.1600-6143.2005.00997.x, PMID: 16095490

[ref92] Yu W. Sandoval R. M. Molitoris B. A. (2007). Rapid determination of renal filtration function using an optical ratiometric imaging approach. Am. J. Physiol. Renal Physiol. 292, F1873–F1880. doi: 10.1152/ajprenal.00218.2006, PMID: 17311910

[ref93] Yvan-Charvet L. Quignard-Boulange A. (2011). Role of adipose tissue renin-angiotensin system in metabolic and inflammatory diseases associated with obesity. Kidney Int. 79, 162–168. doi: 10.1038/ki.2010.391, PMID: 20944545

[ref94] Zhang R. Reisin E. (2000). Obesity-hypertension: the effects on cardiovascular and renal systems. Am. J. Hypertens. 13, 1308–1314. doi: 10.1016/S0895-7061(00)01254-1, PMID: 11130776

[ref95] Zhao X. Khurana S. Charkraborty S. Tian Y. Sedor J. R. Bruggman L. A.. (2017). Alpha Actinin 4 (ACTN4) regulates glucocorticoid receptor-mediated transactivation and Transrepression in Podocytes. J. Biol. Chem. 292, 1637–1647. doi: 10.1074/jbc.M116.755546, PMID: 27998979 PMC5290941

